# Long-term satellite tracking reveals variable seasonal migration strategies of basking sharks in the north-east Atlantic

**DOI:** 10.1038/srep42837

**Published:** 2017-02-20

**Authors:** P. D. Doherty, J. M. Baxter, F. R. Gell, B. J. Godley, R. T. Graham, G. Hall, J. Hall, L. A. Hawkes, S. M. Henderson, L. Johnson, C. Speedie, M. J. Witt

**Affiliations:** 1 Environment & Sustainability Institute, University of Exeter, Penryn Campus, Penryn, Cornwall, TR10 9FE, UK; 2 Centre for Ecology and Conservation, University of Exeter, Penryn Campus, Penryn, Cornwall, TR10 9FE, UK; 3 Scottish Natural Heritage, Silvan House, 231 Corstorphine Road, Edinburgh, EH12 7AT, UK; 4 Department of Environment, Food and Agriculture, Thie Sileau Whallian, Foxdale Road, St John’s, Isle of Man, IM4 3AS; 5 MarAlliance, PO Box 283, San Pedro, Ambergris Caye, Belize; 6 Manx Basking Shark Watch, Glen Chass Farmhouse, Port St Mary, Isle of Man, IM9 5PJ; 7 Scottish Natural Heritage, Great Glen House, Inverness, Scotland, IV3 8NW, UK; 8 Wave Action, 3 Beacon Cottages, Falmouth, TR11 2LZ, UK

## Abstract

Animal migration is ubiquitous in nature with individuals within a population often exhibiting varying movement strategies. The basking shark (*Cetorhinus maximus*) is the world’s second largest fish species, however, a comprehensive understanding of their long-term wider-ranging movements in the north-east Atlantic is currently lacking. Seventy satellite tags were deployed on basking sharks over four years (2012–2015) off the west coast of Scotland and the Isle of Man. Data from 28 satellite tags with attachment durations of over 165 days reveal post-summer ranging behaviours. Tagged sharks moved a median minimum straight-line distance of 3,633 km; achieving median displacement of 1,057 km from tagging locations. Tagged individuals exhibited one of three migration behaviours: remaining in waters of UK, Ireland and the Faroe Islands; migrating south to the Bay of Biscay or moving further south to waters off the Iberian Peninsula, and North Africa. Sharks used both continental shelf areas and oceanic habitats, primarily in the upper 50–200 m of the water column, spanning nine geo-political zones and the High Seas, demonstrating the need for multi-national cooperation in the management of this species across its range.

Animal migration is based upon individuals or groups of individuals attempting to secure optimal environmental conditions and exploit habitats during seasonal changes, and is observed in a wide range of taxa^1^. Some individuals within a population often adopt differing migration strategies, which may result from either inter- or intra-individual plasticity with regards to their fidelity to a particular site. The strength of such fidelity can be affected by food availability, reproductive status, competition, predation risk, or body condition^2^. Describing seasonal and migratory movements in large marine vertebrates can be challenging, largely due to their wide ranging behaviour and the complexities of tracking individuals in water for durations sufficient to observe migratory behaviour^3^. However, advances in satellite tracking technologies and attachment techniques now allow for repeated observations of movements and insights into intra- and inter-individual variation over extended time-scales^4^, enhancing our ability to assess life history traits, distribution and extent of range, site fidelity, migratory movements^4^,^5^,^6^ and exposure to human threat.

Many sharks undertake migrations and utilise resources in different habitats with residency and fidelity varying at different spatial and temporal scales^7^, with further evidence of behavioural plasticity^8^,^9^,^10^,^11^. The basking shark (*Cetorhinus maximus*) is the world’s second largest fish species, historically overexploited for its large liver^12^ resulting in large local population declines leading to recognition by the International Union for Conservation of Nature (IUCN) as Vulnerable globally, and Endangered in the north-east Atlantic^13^; with further designations on a range of conservation legislation in the UK and Europe and inclusion under several international conservation treaties (Table S2). The species has a circumglobal distribution and can undertake extensive trans-oceanic basin migrations^14^,^15^; although the relative frequency and function of these migrations is unknown. Aggregations of basking sharks occur seasonally in temperate continental shelf waters of the Atlantic, Pacific and Indian Oceans to feed, but potentially also for mating and parturition^16^. Population size and structure estimates for the basking shark in the north-east Atlantic are unknown^17^, although a sub-regional estimate has been conducted^18^. Studies in the region have successfully tracked basking sharks for up to 245 days, showing movements into the open ocean, the waters of the Bay of Biscay^19^,^20^ and one trans-Atlantic crossing^14^. These studies however have been limited by sample size, with the majority of movements confined to the continental shelf of the north-east Atlantic (n = 2^14^, n = 7^19^,^21^,^22^, n = 9^20^). With growing concern regarding the rate of decline of global shark populations^23^, the importance of defining the extent and connectivity of mobile species populations has increased^24^.

Basking sharks are considered to be vulnerable to interactions with commercial fishing; potentially becoming entangled in set nets, pot lines or caught incidentally in trawls, and is considered as one of the more valued fins within the shark fin trade^13^.

Anthropogenic activity in the north-east Atlantic is increasing^25^, therefore improved knowledge could be instrumental in supporting management decisions^26^, including mitigation of putative threats such as fisheries bycatch^27^. Area-based protection measures are often implemented based on the majority of individuals exhibiting repeated behaviours and movement patterns. Behavioural plasticity can result in a range of movement strategies, sometimes resulting in groups of individuals moving away from areas originally designated for their protection^9^. These groups may then remain at heightened risk of mortality. Consequently these behaviours may lead to specific groups (potentially based on sex, ages, reproductive status and condition) being at more risk^28^. In this study, long-term movement data gathered from satellite tags attached to basking sharks at known summer ‘hotspots’ off the west coast of Scotland and the Isle of Man^29^,^30^, were used to examine patterns of individual movement and subsequent post-summer migration strategies. Particular attention is given to over-wintering distributions as least is known of basking shark spatial ecology during this period, hence this represents one of the missing links to a more comprehensive understanding of their lifecycle.

## Results

### Satellite tracking

Basking sharks satellite tracked into the year following tag deployment (n = 28) using real-time tags (SPOT; Wildlife Computers) and light-geolocation archival tags (MiniPAT; Wildlife Computers) provided data for a median 281 days (IQR: 247–349; max. 479), moved a median minimum straight-line distance of 3,633 km (IQR 1,987–4,996, range: 469–8,081 km) and were displaced by a median of 1,057 km from their respective tagging locations (IQR: 557–1,384; range: 264–2,711 km). Sharks tracked using SPOTs collected data for a median 322 days (IQR: 252–375; max. 479), moved a median straight-line distance of 2,280 km (IQR: 1,456–3,375; range: 469–4,310 km) and were displaced by a median of 1,057 km from their respective tagging locations (IQR: 374–1,560; range: 264–2,711 km). Sharks tracked using MiniPATs collected data for a median 265 days (IQR: 199–280; max. 292), moved a median straight-line distance of 6,050 km (IQR: 4,044–7,029; range: 2,333–8,081 km) and were displaced by a median of 1,007 km from their respective tagging locations (IQR: 744–1,219; range: 455–2,354 km).

There was no significant interaction effect of sex and estimated body length on the maximum displacement or the minimum latitude recorded by these sharks (GLMM: 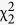  = 5.64, *p* = 0.06 and 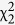 = 5.66, *p* = 0.06 respectively). There were no significant effects of sex, body length or tag attachment duration on the maximum displacement or the minimum latitude recorded by these sharks (GLMM maximum displacement by sex: n = 16, 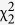 = 1.49, *p* = 0.47; by body length: n = 28, 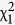 = 0.05, *p* = 0.83 and by tag attachment duration: 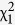 = 0.42, *p* = 0.52. GLMM minimum latitude by sex: n = 16, 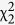 = 0.74, *p* = 0.69; by body length: n = 28, 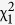 = 0.16, *p* = 0.69 and by tag attachment duration: n = 28, 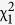 = 0.21, *p* = 0.64. Based on archival tag data, post-summer movements (October onwards) indicated basking sharks entered the Economic Exclusive Zones (EEZs) of Iceland (<1% of all locations), Faroe Islands (2%), UK (18%), Ireland (51%), France (3%), Spain (4%), Portugal (4%), Madeira (<1%), Morocco (<1%), and the High Seas (18%; Fig. S1). Areas of relative high importance for the tracked sharks (Fig. 3) include the waters to the west coast of Scotland, the Celtic and Irish Seas and, in particular the areas west of Ireland along the continental shelf break. These areas experienced a relatively high degree of usage by tracked sharks, somewhat indicative of an overwintering ground that links foraging grounds in the waters off the west coast of the UK and Ireland to the destinations adopted by each of the three migration strategies observed (Fig. 3a).

### Migration strategies

**Figure 1:**
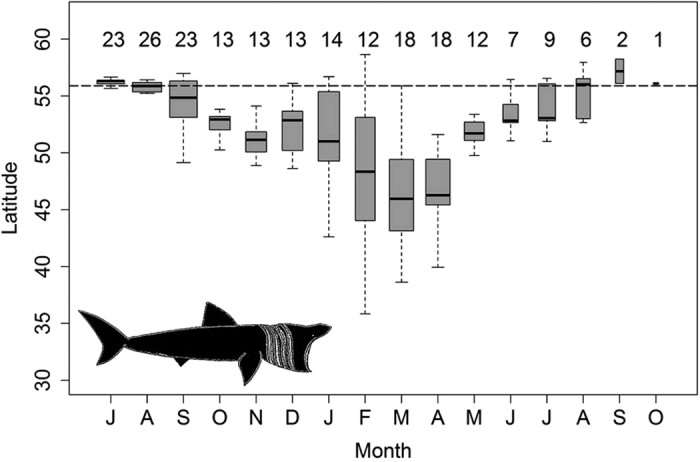
Minimum latitude observed for 28 satellite-tracked basking sharks. Box and whisker plots showing minimum latitudes per shark per month from tag deployment (July onwards). Boxes denote inter-quartile range; horizontal black bar indicates the median (whiskers extend to the 2.5^th^ and 97.5^th^ percentiles). Box width indicates relative data volume of (sample size) for each month; with number of individual sharks contributing to each box shown above corresponding box. Broken line indicates average latitude of tag deployments.

**Figure 2:**
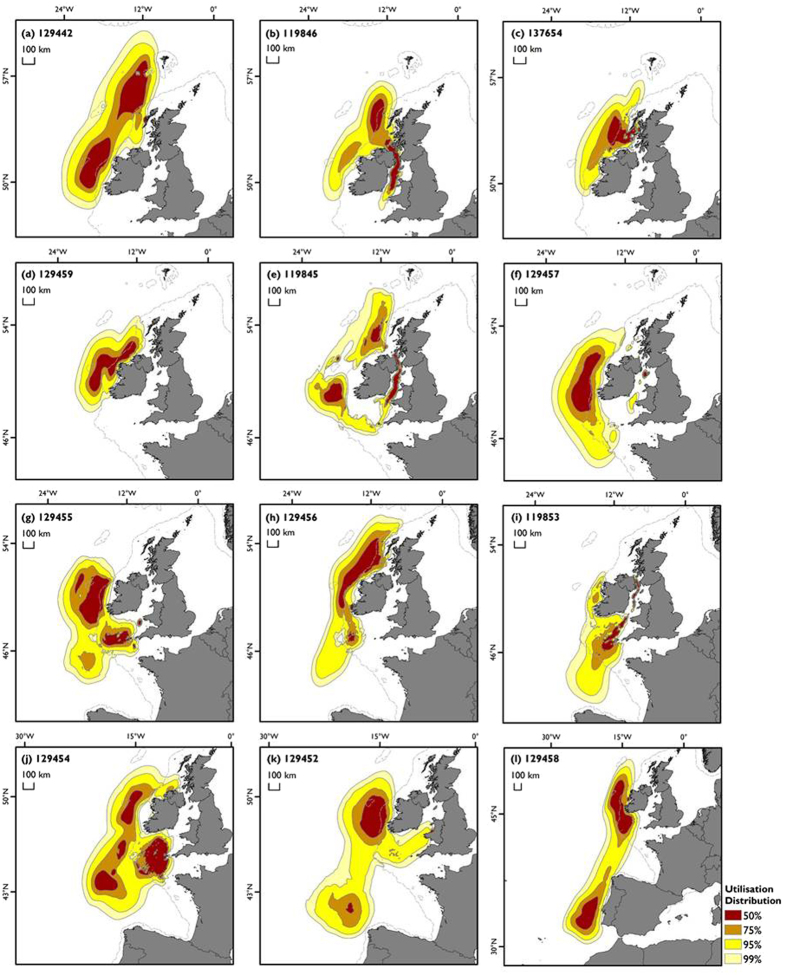
Overall post-summer (October onwards) distribution of individual tracked basking sharks from light-geolocation archival tags (n = 12). Normalised Utilisation Distributions (UDs); shaded according to probability of area of space–use. Broken grey line indicates 200 m depth contour (source: http://www.gebco.net). Maps created in ESRI ArcGIS version 10.1 using ESRI land shapefiles.

**Figure 3:**
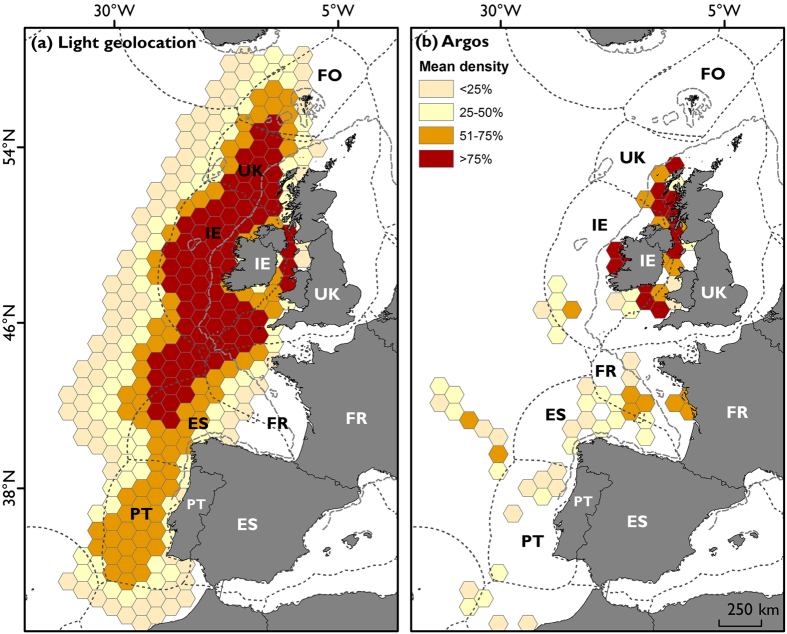
Grid density enumeration identifying areas of relative importance for tracked basking sharks post –summer (October onwards; 2012–2016) for locations derived from light-geolocation archival tags (**a**; n = 12 tags) and Argos real-time tracking tags (**b**; n = 16 tags) on a hexagonal grid (cell edge size: 50 km; cell area: 8,660 km^2^). Country Economic Exclusive Zones denoted by grey broken line with associated international two letter codes (white letters = land, black letters = EEZs; FO = Faroe Islands, UK = United Kingdom, IE = Ireland, FR = France, ES = Spain). Broken dark grey line denotes 200 m depth contour. Maps created in ESRI ArcGIS version 10.1 (http://desktop.arcgis.com/en/arcmap) using ESRI land shapefiles, GEBCO bathymetric contours (http://www.gebco.net) and Flanders Marine Institute (VLIZ) Economic Exclusive Zone (EEZ) boundaries (http://www.marineregions.org).

Basking sharks exhibited wide-ranging post-summer movements, stretching from 33° to 61°N latitude (approx. 3,100 km range) within a longitudinal range (2° to 20°W); along the eastern fringe of the North Atlantic Ocean (Fig. 1 and Figs S2–4). The general pattern of movement followed a transition to more southerly latitudes from October onwards in each year. These movements varied in distance and duration, with some individuals making short-range movements from the tagging areas and others undertaking longer-range movements (Fig. 2 and Figs S2–4). Three post-summer migration strategies were identified from archival tags (n = 12); (a) *Celtic Seas* - predominantly remaining in UK and Ireland, with some movement into waters of the Faroe Islands (n = 6; max. displacement range: 455–854 km; one female, one male, four unknown sex), (b) *Bay of Biscay* - movement south to the Bay of Biscay (n = 5; max. displacement range: 1,161–1,515 km; four females, one male), and (c) *Iberian Peninsula & North Africa* - movement further afield to waters off the west coast of Portugal and North Africa (n = 1; max. displacement: 2,354 km; one unknown sex; Figs S2–4). For Argos Doppler-based geolocation tags (n = 16; three females, six males, seven unknown sex), sharks were displaced by a range of 264–2,711 km.

The furthest movement observed was undertaken by a basking shark during a three-month tracking period using a SPOT tag. This individual departed the west of Scotland tagging area in the month following tag application (August 2012), transited to the west of Ireland and the European mainland and arrived in North African waters in November 2012, at which point the tag ceased transmission (Fig. S4F; minimum straight line along-track distance: 3,949 km, straight line displacement from tagging location: 3,088 km).

### Return migrations

**Figure 4:**
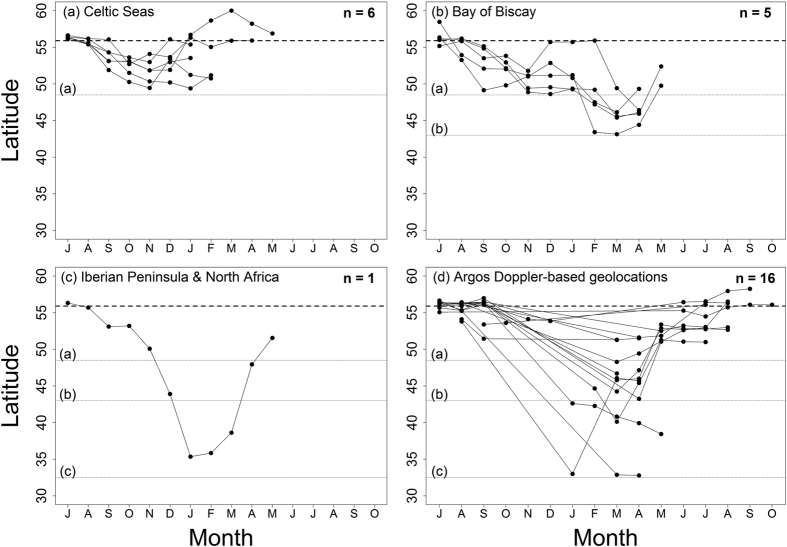
Plots showing minimum monthly latitudes occupied for each tracked shark from tag deployment (July onwards), derived from best daily location estimates from archival tags (n = 12) separated by migration strategy (**a**–**c**) and all Argos Doppler-based geolocation tracked sharks (**d**; n = 16). Minimum latitude for migration strategies (narrow dashed horizontal and labelled lines). Tag deployment locations (thick dashed horizontal line).

We observed varying degrees of return migration (n = 15 tags) in the years following tagging; which can be described as (i) departing the coastal regions of the UK, Isle of Man and Ireland (August to October), and return the following spring/summer (March to June) while remaining within the Exclusive EEZ of the UK and Ireland throughout the winter (Fig. 4a and d, n = 6; tag numbers: 119846 (Fig. S2B), 129439 (Fig. S2C), 129440 (Fig. S2D), 129442 (Fig. S2E), 129457 (Fig. S2G) and 137654 (Fig. S2I)); (ii) movement outside the EEZ of the UK and Ireland during the winter, but return to the Celtic Seas (Fig. 4b and d, n = 3; tag numbers: 129452 (Fig. S3G), 129455 (Fig. S3I) and 129444 (Fig. S4B)); or West Ireland (n = 5; tag numbers: 119853 (Fig. S3A), 129437 (Fig. S3B), 129448 (Fig. S3E), 129456 (Fig. S3J), and 129458 (Fig. S4C)) in spring, having undertaken migration strategy b; *Bay of Biscay* (n = 6; tag numbers: 119853 (Fig. S3A), 129437 (Fig. S3B), 129448 (Fig. S3E), 129452 (Fig. S3G), 129455 (Fig. S3I), and 129456 (Fig. S3J)), or migration strategy c; *Iberian Peninsula & North* Africa (Fig. 4d, n = 2; PTT numbers: 129444 (Fig. S4B) and 129458 (Fig. S4C)); or (iii) full return migration, returning to the region of tag attachment (within approx. 20 km) after over-wintering outside of UK and Irish waters (Fig. 4d, n = 1; PTT number 129449 (Fig. S4F). This is the first observation of such return migration in this species.

### Depth-use

**Figure 5:**
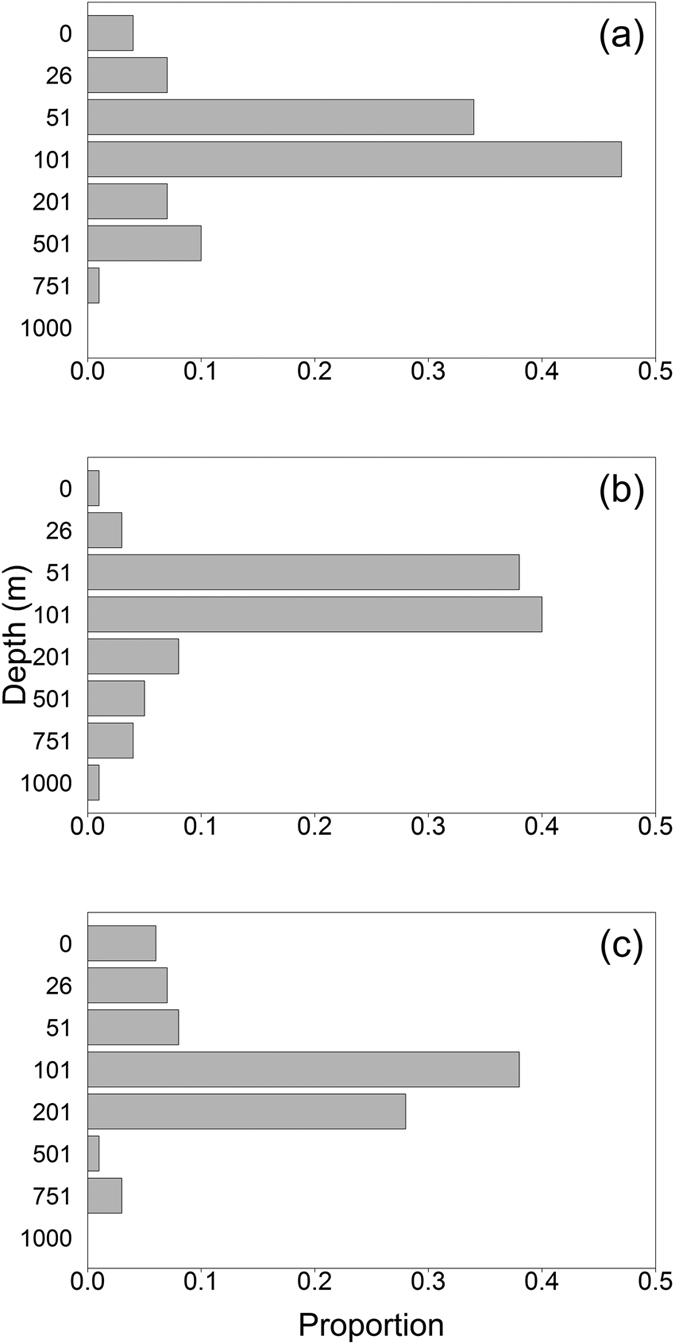
Proportion of daily maximum depths derived from archival tags within eight depth ranges for associated migration strategy; (**a**) Celtic Seas, (**b**) Bay of Biscay and (**c**) Iberian Peninsula and North Africa. Depth ranges are represented by the minimum value for each range (0-25 m, 26–50 m, 51–100 m, 101–200 m, 201–500 m, 501–750 m, 751–1,000 m, >1,000 m).

For those basking sharks tracked with light-geolocation archival tags, data on depth-use were also available. These data highlighted sharks (n = 12) predominantly occupied the epipelagic zone (0–200 m depth; mean 84% of tracking time; Table S5) regardless of migration strategy ((a) *Celtic Seas*: 91%; (b) *Bay of Biscay*: 82%; (c) *Iberian Peninsula & North Africa*: 59%; Fig. 5; Table S5). Individuals exhibiting migration strategy *a* and *b* spent the majority of their time in waters 50–200 m deep (80.2% and 78.2% respectively); whereas, individuals exhibiting migration strategy *c* spent the majority of time in depths between 100 and 500 m (66.2%; Fig. 5; Table S5).

## Discussion

The ability to record intra- and inter-individual variation in the movement and distribution of large marine vertebrates is becoming increasingly possible and provides important information on species space-use^3^,^4^,^5^,^31^, and has resulted in migration being observed in many taxa^1^,^28^. Our study provides the most detailed investigation of basking shark ranging behaviours in the north-east Atlantic over seasonal timescales to be informed by satellite tracking^32^.

Little is known about basking shark habitat or site preference during the winter as their vertical distribution indicates they spend a large proportion of time away from the surface. Anatomical studies previously suggested that basking sharks hibernate in deep waters around the UK and Ireland during the winter^33^,^34^,^35^. In recent years, however, hibernation seems less likely to occur due to increasing levels of information from electronic tags^19^,^36^,^37^. Sims *et al*.^19^ showed that basking sharks do not lie dormant during the winter months, but show frequent vertical movements throughout the water column with close association to the continental shelf edge, providing evidence that these sharks likely do not *hibernate*. More recent studies have shown that this species makes oceanic scale movements post-summer, travelling towards Newfoundland from the Isle of Man^14^, although this has only been observed in a single individual. Extensive north-south autumn migrations have been observed from basking sharks tagged in coastal waters of north-east United States, with tracked individuals crossing the equator into tropical waters off the coast of Brazil^15^. It seems increasingly improbable that this species exhibits a sedentary phase during winter months (based on an assessment of movement), and it remains unknown if basking sharks forage during this time, however, there is evidence for diel vertical migration (DVM) occurring away from the surface post-summer^22^, similar in form to DVM patterns seen in summer months attributed to associating with the diel vertically migrating *Calanus sp.* layer^38^. There is the potential for basking sharks to subsist on fat reserves in the liver, which has been observed in white sharks (*Carcharodon carcharias*) where these sharks exhibited an increased vertical downward drift rate over the course of long migration movements (>4,000 km), which is indicative of decreased buoyancy caused by the depletion of liver lipid reserves^39^. This depletion of lipid reserves has also been noted in historical testimonies from basking shark fishers claiming basking sharks caught earlier in the season had lighter livers^40^.

Historically there have been contrasting opinions on this species’ long-term movements and distribution, with suggestions that basking sharks over-winter as a single population off the coast of North Africa returning northwards in the spring^12^, however, there was a counter argument citing that there was no predictability in first appearance of basking sharks during the spring/summer season from Portugal/Spain northwards as the season progressed^41^. We show that it is unlikely that all basking sharks adopt a single migration strategy, but more likely plasticity occurs within the population, resulting in individuals performing varying movements. It is not yet known whether adopted migration strategy by individuals is annually consistent or changes with body condition, reproductive status, resource availability or other factors.

The primary drivers behind basking shark migrations are still unclear, but may include; searching for foraging grounds, thermoregulation by moving to areas and/or depths of preferred temperature, movement towards mating grounds or natal homing. Skomal *et al*.^15^ hypothesised that within the north-east Atlantic, stable environmental conditions are mediated by the Gulf Stream, limiting the extent to which basking sharks need to move during winter months to find sufficient food. We find that at least some individuals do undertake large-scale latitudinal movements throughout the winter in the north-east Atlantic, somewhat similar to their results from the north-west Atlantic. We have observed the first evidence of round-trip migrations by individuals leaving UK and Irish waters, over-wintering elsewhere, returning to these coastal waters during the spring and summer. Some tracks ended off North Africa with no evidence of return movements, which may be an artefact of tag attachment duration, with premature tag detachment potentially occurring from biofouling of the tag, predation of the tag by other species or removal of the tag during incidental bycatch. There remains the possibility that sharks could move further south, as has been shown in the north-west Atlantic^15^. Shark movements were reconstructed for this study using Argos Doppler-based geolocation and light-geolocation; these techniques differ in that Argos Doppler-based geolocation only provides estimates of locations when the tag is at the surface. During the winter, sharks spend proportionally less time at the surface, limiting opportunities to gather information on their location during this period. In contrast, light geolocation can be near-continuous, particularly when integrated with predictive models of animal movement to provide estimates of location when light geolocation alone is unsuccessful. Our assignment of migration strategy likely underestimates the extent of potential movement for sharks tagged with SPOT tags. Nonetheless, all migration strategies (a to c) were observed independently in the light geolocation data; therefore, broad scale, geographic patterns of movement described here are likely not artefacts of the positioning technology used.

Continued development of tag technology and attachment techniques will allow for multi-year deployments, increasing the ability to quantify individual variability and highlight the likely potential for condition-dependent ranging. Further work is also required to quantify the frequency of newly observed ranging behaviours, whereby individuals adopt a differing behaviour to that of the modal strategy, as these individuals are likely important for maintaining genetic diversity (thought to be low^42^) and ensure the species has the potential to exploit all areas of the realised or fundamental niche^43^,^44^. Greater knowledge on behavioural plasticity may also help improve predictions on how this large planktivorous species might respond to environmental disturbance and climate change, where fidelity to areas may diminish or strengthen as locations that are regularly used by individuals become less suitable, either for foraging or breeding^2^. This may be pertinent for basking sharks, as climate change has been suggested to influence the distribution of their preferred prey group (calanoid copepods^45^,^46^), possibly making some areas less suitable for this species, offering one possible explanation for declines in basking shark sightings within areas of its historical range^47^. Highlighting the full range of movements made by a species and partitioning of time within these areas is integral to implementing effective international conservation measures for highly mobile species^7^,^48^.

In this study, satellite tracked basking sharks largely remained within the EEZs of the UK and Ireland; they also appeared to occupy waters of seven other geo-political zones and the High Seas. In a previous study^49^ it was shown that basking sharks spent a higher proportion of their time in the UK EEZ (31%) to that of our study (18%), however, this study showed a much greater use of the France EEZ (22%) than our study (3%) and much less occupancy of the Ireland EEZ (15%) to that shown here (51%). No use of International waters away from the European continental shelf was shown, whereas we observed basking sharks showing appreciable levels of occupancy of the High Seas (18%). This may be due to shorter tag attachment durations of the previous study, resulting in more data from summer and autumn months. Our study therefore stresses the need for multi-national cooperation in developing a comprehensive conservation strategy for this species, which is still likely recovering from historical exploitation. This is especially apparent during winter months where plasticity in basking shark behaviour results in multiple geo-political zones being occupied by the population and often away from protected areas. Whilst there are no longer targeted fisheries for basking sharks, by-catch is an area of concern, and research in UK waters^50^ has identified incidental catches occurring in fisheries operating off south Ireland in surface and bottom set gill nets^51^,^52^, north-west Iberian Peninsula in artisanal gill net fisheries^53^ and in New Zealand, where basking sharks are a frequent bycatch of trawl and set net fisheries^36^, all with uncertain levels of mortality. The waters to the west of Ireland and the Celtic and Irish Seas are likely important areas for basking sharks, acting as migratory pathways linking foraging areas in the waters off the west coast of Scotland to other areas of importance to basking shark life-history events, which may also include other seasonal foraging or breeding sites. Active fisheries operating within the Irish EEZ, include demersal otter trawling, (approx. 62% of total fishing hours between 2008 and 2012), longliners (15%), gill and trammel nets (7%) and pelagic trawlers (5%) the other most operated gear types^54^. The majority of fishing activity within the Irish EEZ is by foreign vessels (Spanish = 30%, French = 20%, and the UK = 11%), with Irish vessels accounting for 36% of activity with combined landings of over 394,000 tonnes in 2012^54^. The UK is a signatory to the Convention for Migratory Species with Ireland, France, Portugal, Spain and Morocco; all range states for basking sharks, mandating multi-national cooperation over management of shared activities within ranges of species of conservation concern. An onboard bycatch observer programme may provide a useful tool in which to assess the potential impact of bycatch on basking sharks^36^. This would inform on the extent to which basking sharks are being incidentally caught, and provide baseline information on gear type, effort, and potentially mortality rates within these fisheries from which to form an evidence-based conservation programme.

Satellite tracking has greatly improved our understanding of animal movements. This study further contributes to the growing knowledge of basking shark movements and behaviour, especially for those aspects of movement that have remained elusive, such as during winter months in the north-east Atlantic. We show behavioural plasticity within the population, with individuals exhibiting one of three migration strategies and the capacity to move from coastal to oceanic habitats. Individuals can undertake movements at an oceanic scale, crossing multiple geo-political zones following periods of residency. Our work has highlighted a potentially important movement corridor along the continental shelf off western Ireland, which may leave a proportion of the population vulnerable for extended periods to trawl and set-net fishery interactions. We did not detect segregation by sex or size in our study, behaviours that are often reported for sharks^55^,^56^. We cannot fully ascertain whether this is occurring in basking sharks, or whether sample size and access to a full range of sizes and sexes in which to tag affected the results seen. The continued development of tag technology, in particular battery life and minimising biofouling, will allow for longer attachment times, which will increase our understanding of the drivers of movement in this species and intra- and inter-individual movement across multiple years, in order to identify key habitats and behaviours and overlap with potential threats. This research can be coupled with other fast-developing techniques such as stable isotopes and genetic analysis to better estimate population sizes and relatedness and to begin to understand foraging strategies, especially during winter months.

## Methods

Seventy satellite tags (Smart Position or Temperature tags; SPOT = 32; Pop-up Archival Transmitting with Fastloc™ GPS tags; PAT-F = 12; Mini Pop-up Archival Transmitting tag; Mini-PAT = 12; SPLASH-F = 14; Wildlife Computers, Washington, USA) were attached to basking sharks off the west coast of Scotland (n = 62) and Isle of Man (n = 8) during June, July and August in 2012 (n = 21), 2013 (n = 36), 2014 (n = 10) and 2015 (n = 3)^32^ (for tag programming and deployment see supplementary materials). The attachment of satellite transmitters in Scottish coastal waters protocol was approved by the UK HM Government Home Office under the Animals (Scientific Procedures) Act 1986 (issuing Project Licence 30/2975). All work was carried out in accordance with the UK HM Government Home Office under the Animals (Scientific Procedures) Act 1986 (Project Licence 30/2975) and under the Wildlife & Countryside Act 1981 (as amended) (Licence(s): 13904, 13937 and 13971) and internally through the University of Exeter’s animal welfare and ethics review board (AWERB). Licences to tag sharks in the Isle of Man were issued by the Department of Environment, Food and Agriculture (Isle of Man Government) under the Wildlife Act 1990. Data gathered from 29 sharks (SPOT = 16; PAT-F = 3; Mini-PAT = 8; SPLASH-F = 2) were selected for detailed analysis; these sharks were either tracked into at least the January following tag attachment (n = 28; >165 days of tracking; Table S2), or were tracked making long-range movements away from the north-east Atlantic over a shorter period of time (n = 1; Table S2). All tag data were downloaded from CLS-Argos and archived using the Satellite Tracking and Analysis Tool (STAT)^57^. Basking sharks were geolocated during their tracking periods using either standard Argos Doppler-based geolocation when sharks were at the surface (n = 16; SPOT and SPLASH-F tags) or light-based geolocation throughout the tag attachment period (n = 12; PAT-F, Mini-PAT and SPLASH-F tags). These data were subsequently processed to single daily tracking locations for each individual. Argos Doppler-based geolocation filtering was achieved using the *adehabitat* package^58^.

Light geolocation data were obtained from archival tags (n = 12, one SPLASH-F tag failed to transmit sufficient light level data for track reconstruction) and analysis of light level data was undertaken by *Collecte Localisation Satellites* (CLS-Argos) (www.argos-system.org). Obtaining daily estimates of location from gathered light data can be challenging for basking sharks as they often spend prolonged periods at depth or exhibit diel vertical migration (DVM), reducing reliability of some light data^22^. Therefore, to reconstruct the likely movement paths of basking sharks, we used Hidden Markov Models (HMM) implemented as grid filters^59^ to estimate the daily probability density (or Utilisation Distribution; UD) of the location of tracked animals making use of validated light-based estimates of location to influence the resulting modelled trajectories^60^. The HMM used a two-step process, whereby at each sampling time a position prediction step, solving the advection-diffusion equation for the two-dimensional probability of an animal’s presence, was implemented^61^. An update step was then performed to combine the predicted probability density using information on latitude, longitude, SST (GHRSST-OSTIA; https://www.ghrsst.org/) and depth (etopo2; https://www.ngdc.noaa.gov/mgg/global/etopo2.html) recorded onboard the tag to produce the posterior distribution of the individual^61^. Locations derived from light intensity (obtained using Wildlife Computers GPE2 software) were used as observations. These data were constrained by bathymetry^60^, SST and known deployment and pop-off locations. The diffusion coefficient of the HMM model was set to 1,000 km^2^d^−1^; the standard deviation of raw light based locations used in the update step was set to 1° longitude and 3.5° latitude and the standard deviation of the difference between recorded and satellite derived SST was set to 0.5 °C^61^. The best daily estimate of location for these tags was taken to be the geographic mean of the grid locations weighted by their probability. Once daily UDs were calculated for each tag for the duration of the tag attachment, these were normalised and summed to provide the probability of the animal’s presence in the extent of the grid filter for its time at liberty. For each daily distribution probability raster, percentage volume contours (PVC) were calculated to produce density kernels exhibiting likelihood of presence (Fig. 2). UDs for each shark were created for entire time at liberty post-summer (October onwards). Data from PAT-F, MiniPAT and SPLASH-F tags recording depth (n = 12) were used to estimate time spent within pre-determined depth ranges.

To determine areas of high relative importance for tracked basking sharks polygon sampling grids bounded by the maximum limits of observed movement were spatially intersected with filtered tracking locations for Argos Doppler-based geolocation and raster values for light-based geolocation (hexagonal cells; 50 km from grid cell centroid to edge; cell area 8,660 km^2^). The size of the grid cells was based on the mean error across all light-based geolocation tags (97.68 km). The mean occurrence of daily locations within grid cells was calculated for each individual followed by a spatial mean calculated across all individuals. All spatial analyses and maps were created using Geospatial Modelling Environment (GME v 0.7.2.1)^62^ and ESRI ArcMap 10.1.

K-means cluster analysis was used to separate individual tracks into migration strategy groups^63^ based on most southerly latitude observed using best daily locations, which was used as a proxy for putative migration strategy. This analysis was conducted using archival tags only (n = 12), as data provided information on the full extent of movement with robust evidence of most southerly latitude reached, followed by return movements North in the spring. All data analyses were performed in R^64^.

To examine the effect of basking shark sex, body length and tag attachment duration on movement we used General Linear Mixed-effect Modelling (GLMMs; *lme4* package^65^). For this analysis the maximal model was fitted with all biologically relevant interactions. The significance of fixed effects were assessed by comparing maximum likelihood ratios of the maximal model to the model without the fixed effect, with non-significant interactions removed to test the main effects^66^.

## Additional Information

**How to cite this article**: Doherty, P. D. *et al*. Long-term satellite tracking reveals variable seasonal migration strategies of basking sharks in the north-east Atlantic. *Sci. Rep.* **7**, 42837; doi: 10.1038/srep42837 (2017).

**Publisher's note:** Springer Nature remains neutral with regard to jurisdictional claims in published maps and institutional affiliations.

## Supplementary Material

Supplementary Materials
