# Supplementary material for: Long-term satellite tracking reveals variable seasonal migration strategies of basking sharks in the north-east Atlantic
Source: Sci Rep. 2017 Feb 20;7:42837. doi: 10.1038/srep42837 (PMC5316944; doi:10.1038/srep42837)
Supplement: Supplementary Materials [file srep42837-s1.doc]

**Long-term satellite tracking reveals variable seasonal migration strategies of basking sharks in the north-east Atlantic**

**Doherty, P.D.^1,2^, Baxter, J.M.^3^, Gell, F.R.^4^, Godley, B.J.^1,2^, Graham, R.T.^5^, Hall, G.^6^, Hall, J.^6^, Hawkes, L.A.^2^, Henderson, S.M.^7^, Johnson, L.^8^, Speedie, C.^8^, & Witt, M.J.^1, 2*^**

^1^Environment & Sustainability Institute, University of Exeter, Penryn Campus, Penryn, Cornwall, TR10 9FE, UK.

^2^Centre for Ecology and Conservation, University of Exeter, Penryn Campus, Penryn, Cornwall, TR10 9FE, UK.

^3^Scottish Natural Heritage, Silvan House, 231 Corstorphine Road, Edinburgh, EH12 7AT, UK.

^4^Department of Environment, Food and Agriculture, Thie Sileau Whallian, Foxdale Road, St John’s, Isle of Man, IM4 3AS.

^5^MarAlliance, PO Box 283, San Pedro, Ambergris Caye, Belize.

^6^Manx Basking Shark Watch, Glen Chass Farmhouse, Port St Mary, Isle of Man, IM9 5PJ.

^7^Scottish Natural Heritage, Great Glen House, Inverness, Scotland, IV3 8NW, UK.

^8^Wave Action, 3 Beacon Cottages, Falmouth, TR11 2LZ, UK.

*Corresponding author.

**Supplementary materials**

**Methods**

The attachment of satellite transmitters in Scottish coastal waters protocol was approved by the UK HM Government Home Office under the Animals (Scientific Procedures) Act 1986 (issuing Project Licence 30/2975). All work was carried out in accordance with the UK HM Government Home Office under the Animals (Scientific Procedures) Act 1986 (Project Licence 30/2975) and under the Wildlife & Countryside Act 1981 (as amended) (Licence(s): 13904, 13937 and 13971) and internally through the University of Exeter’s animal welfare and ethics review board (AWERB). Licences to tag sharks in the Isle of Man were issued by the Department of Environment, Food and Agriculture (Isle of Man Government) under the Wildlife Act 1990. Sharks were approached with a boat from behind to avoid the shark’s line of sight and to minimise disturbance. On approach to the sharks, the individuals were, where possible, sexed (female (n = 8), male (n = 8), and unknown (n = 12) using a pole mounted camera and total body length estimated (4-5 m (n = 6), 5-6 m (n = 12), 6-7 m (n = 5), 7-8 m (n = 4) and 8-9 m (n = 1) based on comparison to the length of the survey boat (10 m). Tags were deployed using a titanium M-style dart (Wildlife Computers) inserted into the sub-dermal layer at the base of the first dorsal fin with a modified pole spear and attached via a tether consisting of heat-shrink covered stainless steel flexible cable, a swivel and monofilament line attached to the satellite tag.

Four models of satellite tags were deployed; Smart Position or Temperature tags (SPOT; n = 32), transmitting location data when at the surface via the ARGOS satellite system. Pop-up Archival Transmitting with Fastloc™ GPS tags (PAT-F; n = 12), Mini Pop-up Archival Transmitting tags (Mini-PAT; n = 12) and SPLASH-F archival tags (n = 14). All archival tags collected light, temperature and depth data at 10 second (PAT-F) or 15 second (Mini-PAT & SPLASH-F) intervals. Throughout the project, satellite tags were attached to a total of 24 females, 19 males and 27 individuals of unknown sex, measuring 4-5m (n = 10), 5-6m (n = 30), 6-7m (n = 14), 7-8m (n = 13) and 8-9m (n = 3) estimated total length. Tags were programmed to record summarised percentage depth use across 12 depth ranges; 0-1m, 1-5m, 5-10m, 10-25m, 25-50m, 50-75m, 75-100m, 100-250m, 250-500m, 500-750m, 750-1000m and >1000m with this information created every four hours. Satellite transmitted archival maximum daily depths were used to estimate location within the water column during wide-ranging movements.

Location data from SPOT tags were subject to filtering, leaving only location classes 1 (accurate to 500-1500m), 2 (accurate to 250-500m), 3 (accurate to <250m), ‘A’ (three messages received but no accuracy estimation) and ‘B’ (one or two messages received but no accuracy estimation )[33]. A maximum plausible speed filter was applied removing locations if speed between two locations exceeded 10 km h^-1^. These data were later reduced to a single, most accurate best daily location to minimise spatial and temporal autocorrelation.

Satellite tracking end points were determined by a pre-determined tag detachment date or earlier due to tag attachment failure or other unknown factors leading to detachment of tags. When an Argos Doppler derived location tag detaches from the study animal, it will float on the surface generating many high quality locations over several days. These data are unusual for tags attached to wild animals, as it does not reflect their natural behaviour, and as such provides a useful indicator that the tag has detached.

**Table S1. Legislation**. National and international regulations and protection measures for basking sharks.

| **Regulation** | **Year** | **Region** |
| --- | --- | --- |
| Wildlife & Countryside Act | 1981 | UK |
| Manx Wildlife Act | 1990 | Isle of Man |
| Biodiversity Action Plan (BAP) priority species | 1997 | UK |
| Countryside Rights of Way Act | 2000 | UK |
| CITES (Appendix II) | 2003 | Global |
| CMS (Appendix I & II) | 2005 | Global |
| European Common Fisheries Policy (EU CFP) | 2007 | Europe |
| OSPAR Convention for the Protection of the Marine Environment of the  North-East Atlantic: OSPAR List of Threatened and/or Declining Species and Habitats | 2008 | Europe |
| Marine & Coastal Access Act | 2009 | UK |
| Marine (Scotland) Act | 2010 | Scotland |
| Marine Act (Northern Ireland) | 2013 | Northern Ireland |

**Table S2. Deployment information**. Summary table of tags remaining attached for longer than 165 days, displaying ‘over-wintering’ behaviour used in this study (n = 28), plus one SPOT tracked sharks included for demonstration of behaviours at different time-scales. Ordered by deployment year and tag attachment duration.


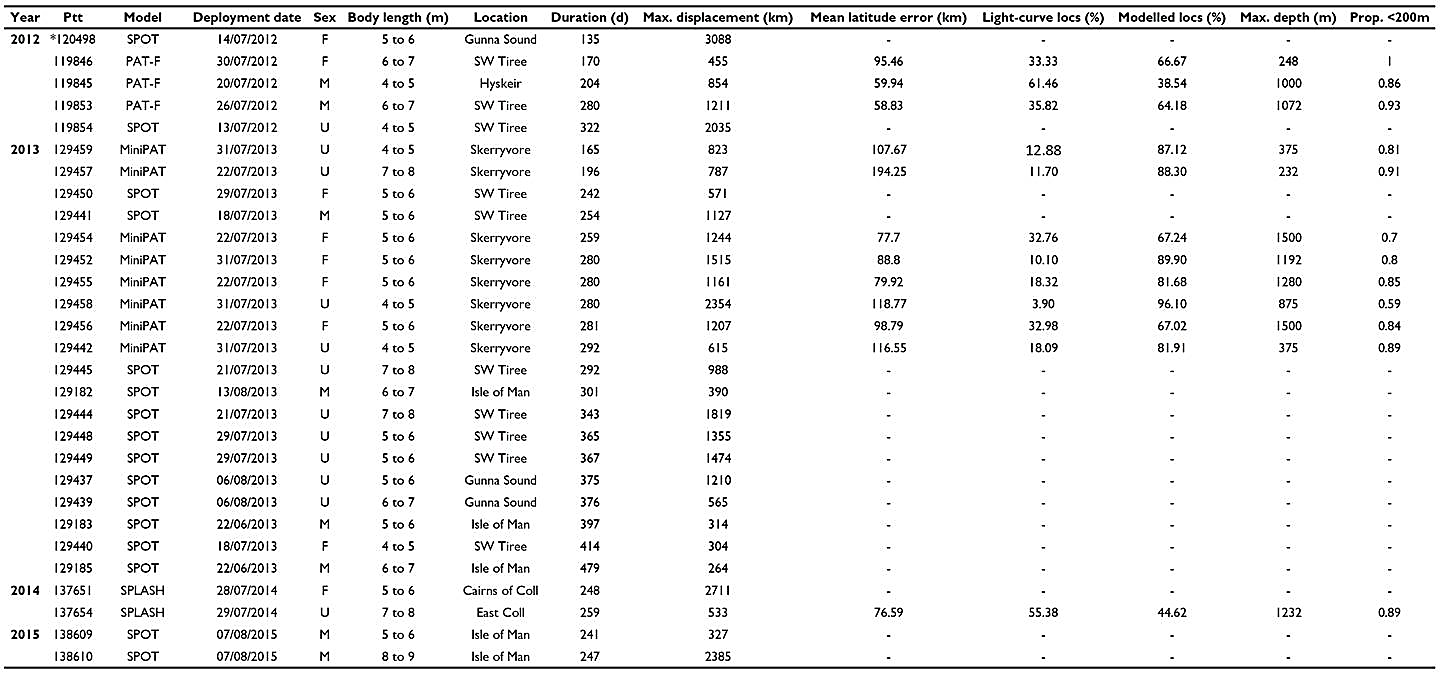


*Does not meet tracking duration requirements for analysis, included for behaviour demonstrative purposes.

Sex: M = male, F = female & U = unknown.

**Table S3. Use of geo-political marine zones in the north-east Atlantic**. Proportions of density values from daily distribution utilisation distributions within each sovereign state’s marine boundaries. Table ordered by EEZ from most to least densely occupied zone.

| **EEZ** | **Prop** | **%** |
| --- | --- | --- |
| Ireland | 0.51 | 50.65 |
| UK | 0.18 | 18.10 |
| High seas | 0.18 | 17.58 |
| Spain | 0.04 | 4.26 |
| Portugal | 0.04 | 3.57 |
| France | 0.03 | 3.41 |
| Faeroe Islands | 0.02 | 1.96 |
| Morocco | <0.01 | 0.23 |
| Iceland | <0.01 | 0.13 |
| Madeira | <0.01 | 0.10 |

**Table S4. K-m**eans cluster analysis criteria.

| **Year** | **Ptt** | **Min. Latitude** | **Migration strategy** |
| --- | --- | --- | --- |
| **2012** | 119846 | 52.98 | A |
|  | 119845 | 49.45 | A |
|  | 119853 | 45.56 | B |
| **2013** | 129459 | 51.85 | A |
|  | 129457 | 49.38 | A |
|  | 129454 | 45.41 | B |
|  | 129452 | 43.14 | B |
|  | 129455 | 46.13 | B |
|  | 129458 | 35.35 | C |
|  | 129456 | 46.41 | B |
|  | 129442 | 51.84 | A |
| 2014 | 137654 | 52.70 | A |

**Table S5. Depth-use of satellite tracked basking sharks for post-summer (October onwards) movements.** Proportions of locations received within specific depth classes. Separated by assigned migration strategy.

| ***(a) Celtic Seas*** |  |  |
| --- | --- | --- |
| **Depth class** | **No. Days** | **Prop.** |
| 0-25 | 34 | 0.04 |
| 26-50 | 57 | 0.07 |
| 51-100 | 293 | 0.34 |
| 101-200 | 411 | 0.47 |
| 201-500 | 58 | 0.07 |
| 501-750 | 5 | 0.01 |
| 751-1000 | 11 | 0.01 |
| >1000 | 4 | 0 |
| **Total** | **873** |  |
|  |  |  |
| ***(b) Bay of Biscay*** |  |  |
| **Depth class** | **No. Days** | **Prop.** |
| 0-25 | 8 | 0.01 |
| 26-50 | 34 | 0.03 |
| 51-100 | 379 | 0.38 |
| 101-200 | 394 | 0.4 |
| 201-500 | 78 | 0.08 |
| 501-750 | 47 | 0.05 |
| 751-1000 | 36 | 0.04 |
| >1000 | 12 | 0.01 |
| **Total** | **988** |  |
|  |  |  |
| ***(c) Iberian Peninsula & North Africa*** |  |  |
| **Depth class** | **No. Days** | **Prop.** |
| 0-25 | 4 | 0.06 |
| 26-50 | 5 | 0.07 |
| 51-100 | 6 | 0.08 |
| 101-200 | 27 | 0.38 |
| 201-500 | 20 | 0.28 |
| 501-750 | 7 | 0.10 |
| 751-1000 | 2 | 0.03 |
| >1000 | 0 | 0.00 |
| **Total** | **71** |  |


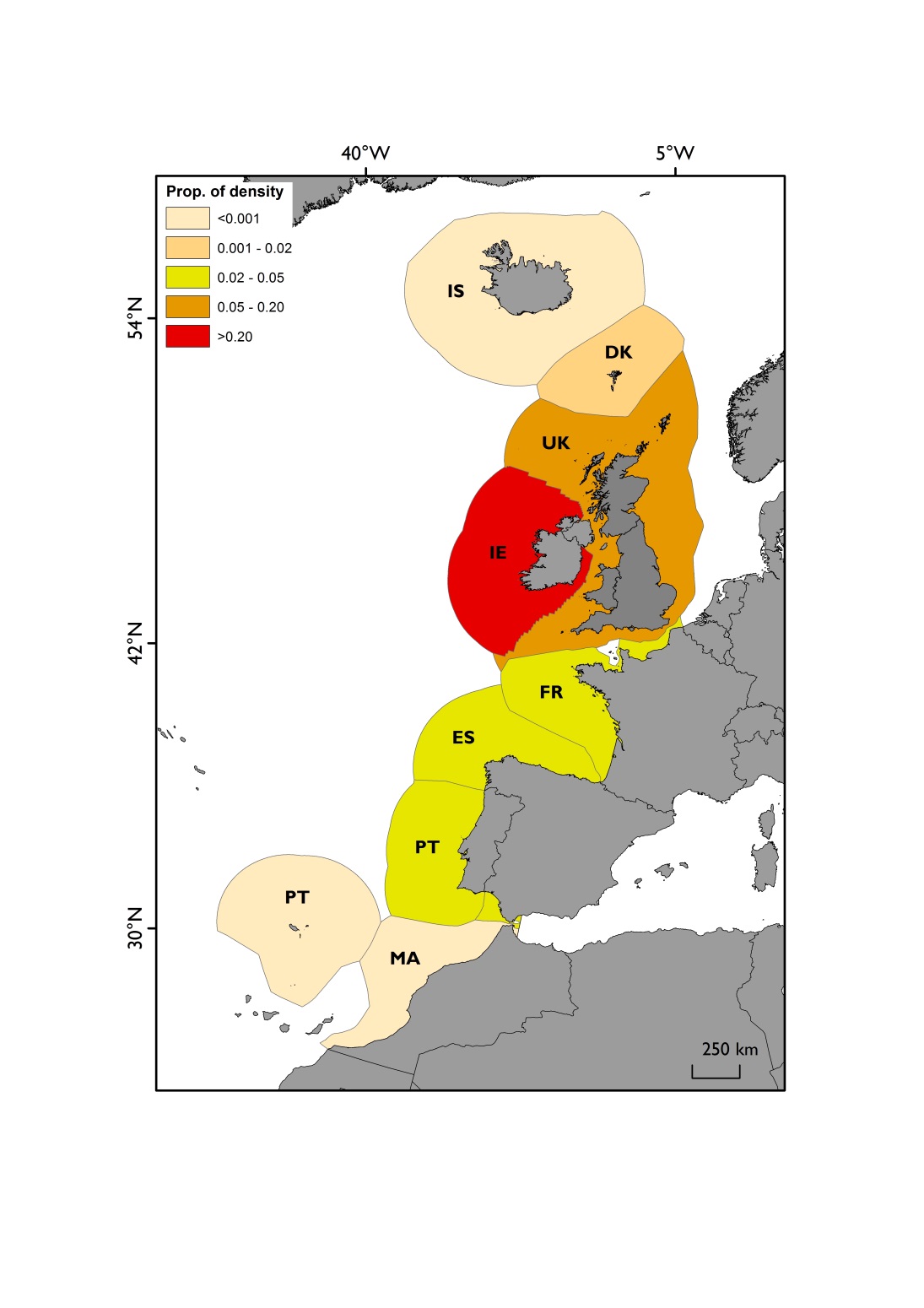


**Fig S1. Geo-political zone use by tracked basking sharks.** The north-east Atlantic split into Economic Exclusive Zones (EEZs) for each country in which tracking locations were received for satellite tracked basking sharks. Each EEZ coloured according to proportion of occupancy within its boundaries. EEZs labelled with international two letter initials for sovereign state of each region (IS=Iceland, DK=Denmark, UK=United Kingdom, IE=Ireland, FR=France, ES=Spain, PT=Portugal and MA=Morocco). Map created in ESRI ArcGIS version 10.1 (Http://desktop.arcgis.com/en/arcmap) using Esri land shapefiles and Flanders Marine Institute (VLIZ) Economic Exclusive Zone (EEZ) boundaries (http://www.marineregions.org).


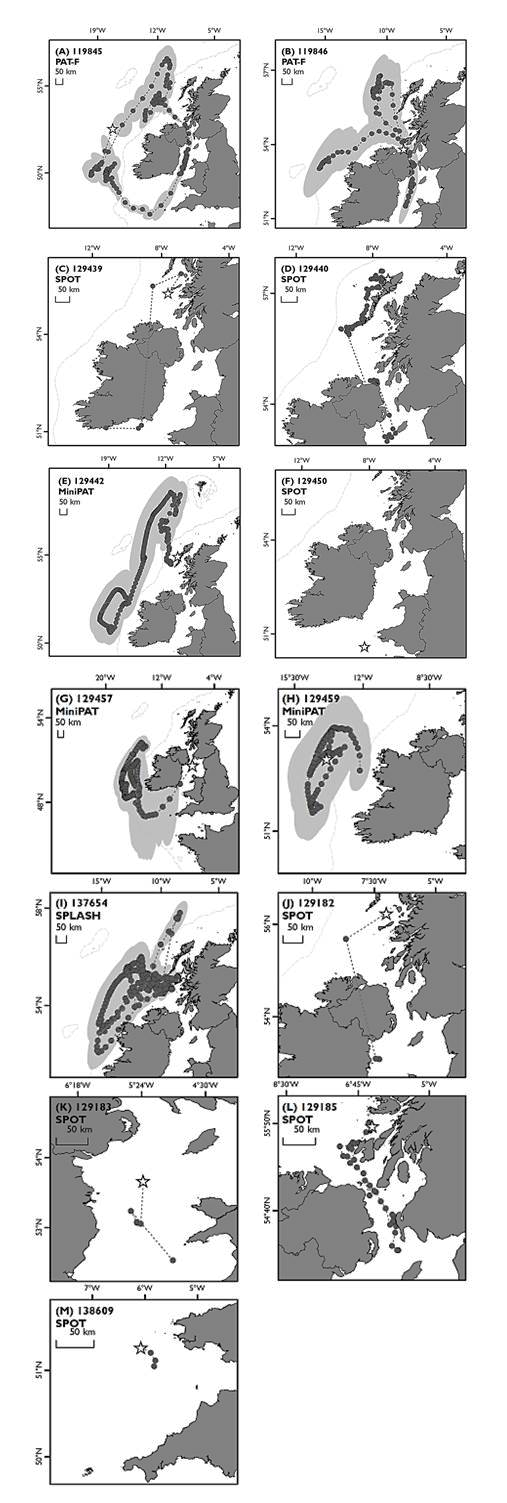


**Fig S2. Individual movements of basking sharks** **exhibiting migration strategy *A* (*Celtic Seas*).** Satellite tracked data from basking sharks for post-summer (October onwards) movements. Each circle represents best daily location, with associated error from light geolocation displayed as grey ellipses. Shark tag ID and tag model type displayed. Note figure parts are to differing scales. Broken grey line denotes 200 m bathymetric contour. White stars denote track end point for Argos Doppler-based geolocation tags or pop-off locations for light geolocation tags. Maps created in ESRI ArcGIS version 10.1 (http://desktop.arcgis.com/en/arcmap) using ESRI land shapefiles and GEBCO bathymetric contours (http://www.gebco.net).


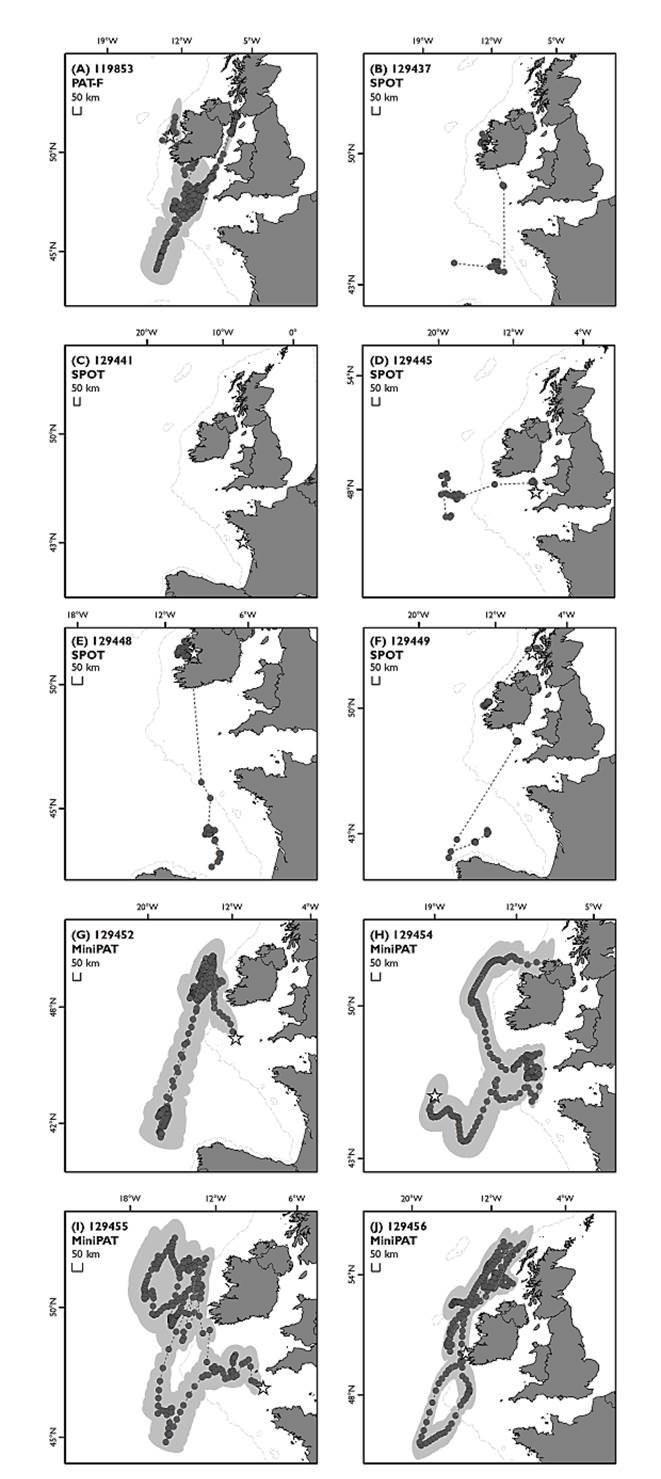


**Fig S3. Individual movements of basking sharks exhibiting migration strategy *B* (*Bay of Biscay*).** Satellite tracked data from basking sharks for post-summer (October onwards) movements. Each circle represents best daily location, with associated error from light geolocation displayed as grey ellipses. Shark tag ID and tag model type displayed. Note figure parts are to differing scales. Broken grey line denotes 200 m bathymetric contour. White stars denote track end point for Argos Doppler-based geolocation tags or pop-off locations for light geolocation tags. Maps created in ESRI ArcGIS version 10.1 (http://desktop.arcgis.com/en/arcmap) using ESRI land shapefiles and GEBCO bathymetric contours (http://www.gebco.net).

**
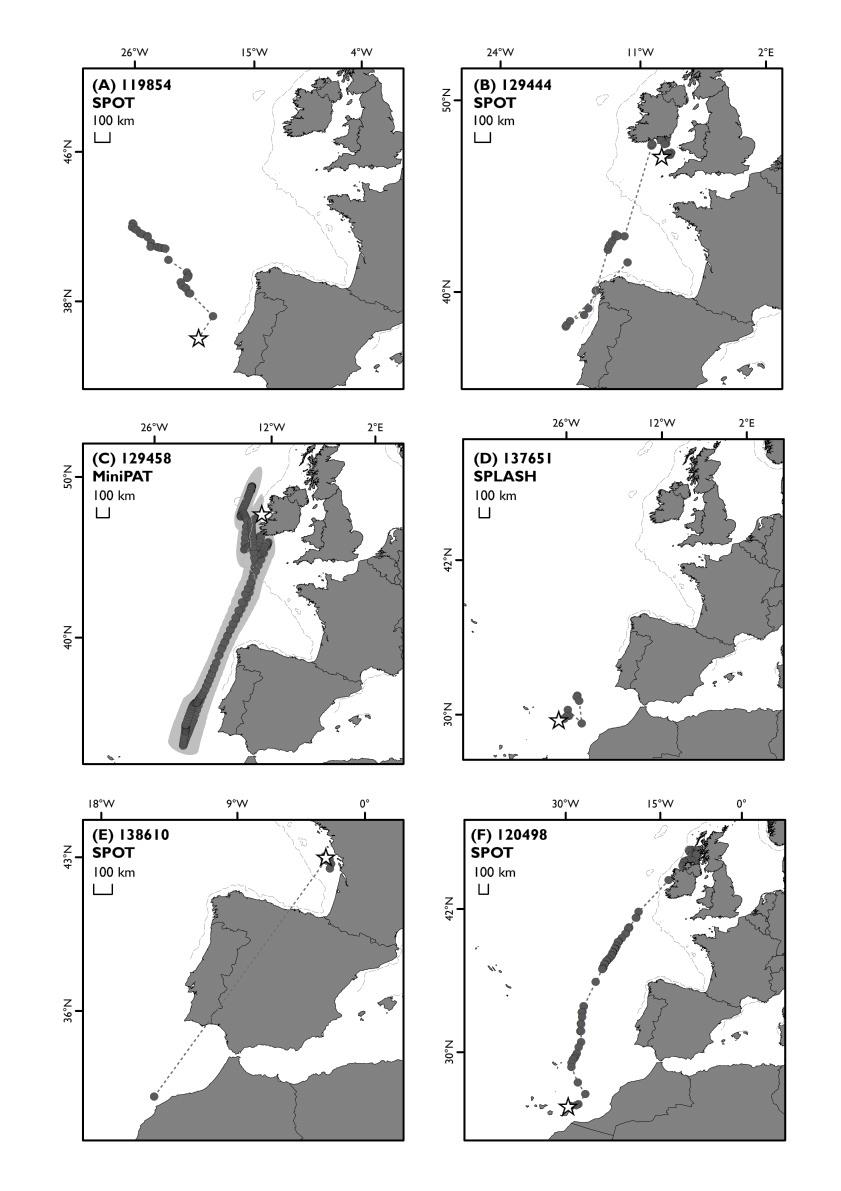
**

**Fig S4. Individual movements of basking sharks exhibiting migration strategy *C* (Iberian Peninsula & North Africa).** Satellite tracked data from basking sharks for post-summer (October onwards) movements, with additional shark tracked in 2012 exhibiting migration strategy *C* on a shorter time-scale (F). Each circle represents best daily location, with associated error from light geolocation displayed as grey ellipses. Shark tag ID and tag model type displayed. Note figure parts are to differing scales. Broken grey line denotes 200 m bathymetric contour. White stars denote track end point for Argos Doppler-based geolocation tags or pop-off locations for light geolocation tags. Maps created in ESRI ArcGIS version 10.1 (http://desktop.arcgis.com/en/arcmap) using ESRIESRI land shapefiles and GEBCO bathymetric contours (http://www.gebco.net).
